# High-throughput Analysis of Capillary Density in Skeletal Muscle Cross Sections

**DOI:** 10.21769/BioProtoc.4922

**Published:** 2024-01-20

**Authors:** Tooba Abbassi-Daloii, Sander D. Mallon, Salma El Abdellaoui, Lenard M. Voortman, Vered Raz

**Affiliations:** 1 Human Genetics, Leiden University Medical Centre, Leiden, The Netherlands; 2 Department of Bioinformatics-BiGCaT/NUTRIM, Maastricht University, Maastricht, The Netherlands; 3 Cell and Chemical Biology, Leiden University Medical Centre, Leiden, The Netherlands

**Keywords:** Capillary density, Image quantification, Skeletal muscle, Immunofluorescence, CD31, Endoglin

## Abstract

Capillary density in skeletal muscles is key to estimate exercise capacity in healthy individuals, athletes, and those with muscle-related pathologies. Here, we present a step-by-step, high-throughput semi-automated method for quantifying capillary density from whole human skeletal muscle cross-sections, in areas of the muscle occupied by myofibers. We provide a detailed protocol for immunofluorescence staining, image acquisition, processing, and quantification. Image processing is performed in ImageJ, and data analysis is conducted in R. The provided protocol allows high-throughput quantification of capillary density.

Key features

• This protocol builds upon the method and results described in Abbassi-Daloii et al. (2023b).

• It includes step-by-step details on image acquisition and image processing of the entire muscle section.

• It enables high-throughput and semi-automated image quantification of capillary density.

• It provides a robust analysis for determining capillary density over the entire muscle cross section.

**Graphical overview**


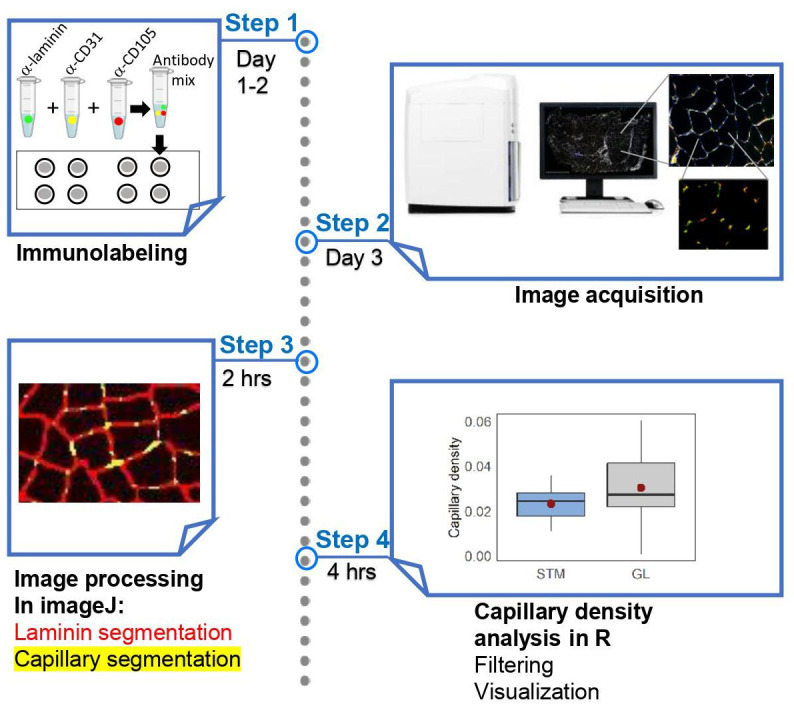


## Background

Capillaries in skeletal muscles play a vital role in the delivery of oxygen and nutrients essential for muscle metabolism and contraction, both at rest and during exercise. Capillary density, which refers to the number of capillaries in a given myofiber area, is critical for estimating oxygen consumption and determining exercise capacity in athletes, the elderly, and patients with muscle-related pathologies. Low capillary density in skeletal muscles is an indicator of reduced oxidative metabolism (Duscha et al., 2020). Conversely, a higher capillary density shortens the distance for oxygen diffusion, leading to improved muscle performance (Gliemann, 2016). Importantly, capillary density can adapt to different conditions and stimuli. For example, endurance training increases muscle capillary density, whereas physical or medical conditions associated with muscle disuse can negatively affect capillary density (Lemieux and Birot, 2021). Thus, determining capillary density is a key measure for assessing changes in skeletal muscle physiology and evaluating the exercise potential of skeletal muscles.

**Figure 1.**
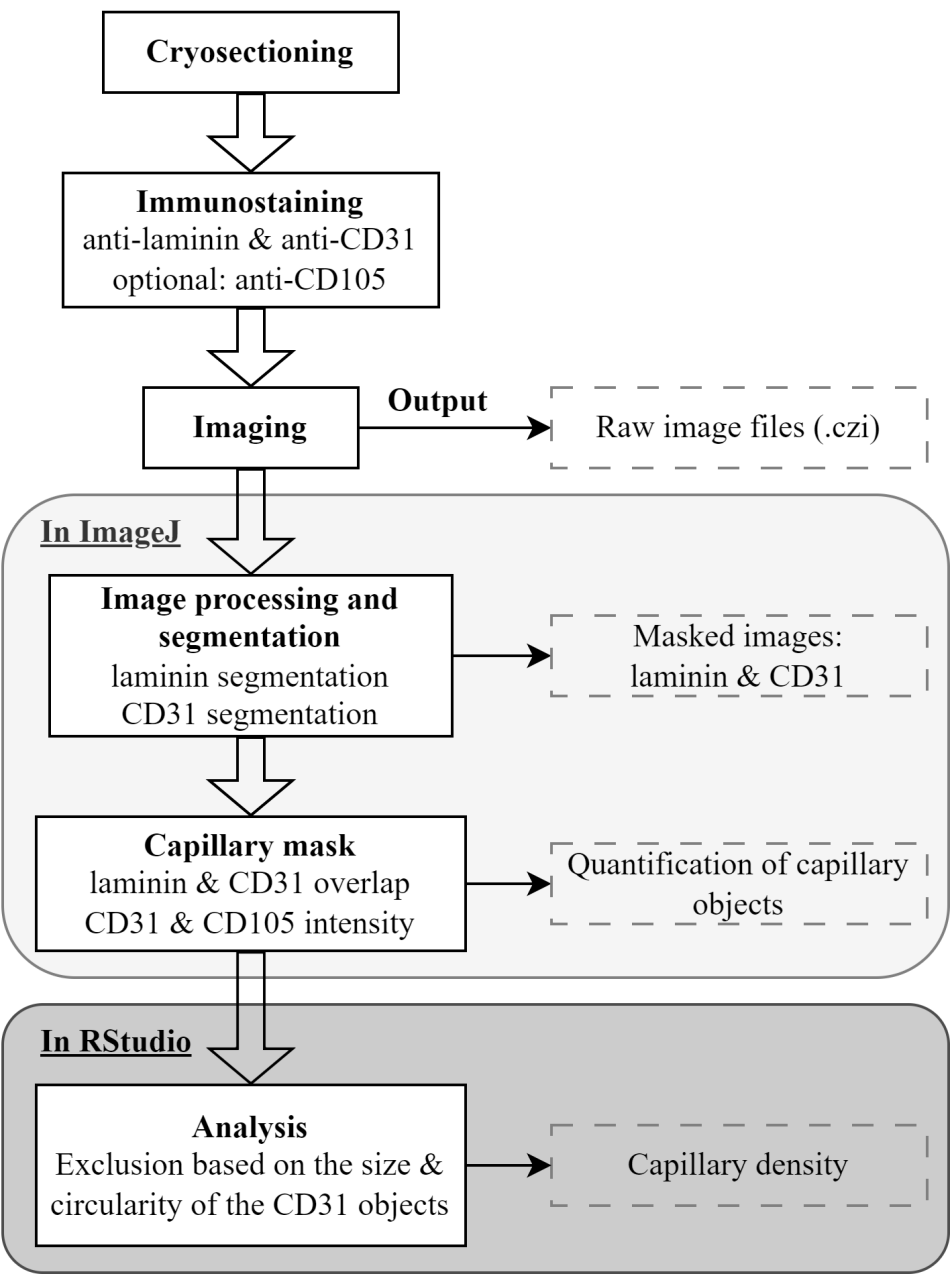
Flowchart of the main steps in the protocol. In the immunostaining step, an anti-laminin antibody marks the cell membrane, while anti-CD31 and anti-CD105 antibodies mark epithelial cells.

Capillary density is defined as the number of capillaries per unit of muscle cross-sectional area in a muscle biopsy (McGuire and Secomb, 2003; Abbassi-Daloii et al., 2023b). This only takes into account the myofibers within the muscle tissue, and therefore excludes fibrotic regions. Determination of capillary density is essential for estimating oxygen consumption and blood flow in skeletal muscles. It involves immunohistochemistry in muscle cross sections using antibodies specific for proteins expressed in endothelial cells, such as CD31 and/or CD105 (endoglin) (Pestronk et al., 2010; Duscha et al., 2020). In some protocols, capillaries are stained with *Ulex europaeus* agglutinin (Hendrickse et al., 2022), which stains lectins (N-glycans) and is used as a marker for endothelial cells (Holthöfer et al., 1982). Most protocols for measuring capillary density rely on manual, eye-based evaluation of fluorescence-stained muscle tissue, as shown in examples such as Andersen (1975), Duscha et al. (2020), and Baum et al. (2023). However, eye-based image scoring has limitations, due to its susceptibility to bias, time-consuming nature, and low throughput, resulting in reduced reproducibility and less robust results. Alternative procedures use image quantification, but these are also low throughput and cover only a small part of the muscle cross-section, leading to a spatial bias (Hendrickse et al., 2022). High-throughput semi-automated imaging and image quantification of the entire muscle cross section overcomes these limitations. We recently reported on a large study of human skeletal muscles that required high-throughput imaging and image analysis of immunohistochemistry in skeletal muscles (Abbassi-Daloii et al., 2023b). While we have previously presented a high-throughput protocol for myofiber typing (Abbassi-Daloii et al., 2023a), here, we present a high-throughput protocol for assessing capillary density in skeletal muscles. A flowchart summarizing the steps implemented in this protocol is shown in Figure 1.

## Materials and reagents

**Biological materials**

1. Snap-frozen human skeletal muscle biopsy

**Reagents**

**Antibodies**

1. Anti-human CD31-Alexa Fluor^®^ 594-conjugated; dilution 1:400 (BioLegend, catalog number: 303126)
2. Rabbit anti-laminin; dilution 1:2,000 (Sigma-Aldrich, catalog number: L9393)
3. Goat anti-rabbit Alexa Fluor^®^ 750-conjugated; dilution 1:1,000 (Thermo Fisher Scientific, catalog number: A21039)
  Optional:
4. Anti-human CD105 (endoglin, ENG) biotin-conjugated; dilution 1:100 (BioLegend, catalog number: 323214)
5. Streptavidin-Alexa Fluor^®^ 647-conjugated; dilution 1:500 (Life Technologies, catalog number: S21374)

**Chemicals**

1. OCT Embedding matrix for frozen sections (Tissue-Tek) (VWR, part of Avantor, catalog number: 361603E)
2. NaCl (Sigma-Aldrich, catalog number: 7647-14-5)
3. Na_2_HPO_4_·2H_2_O (Sigma-Aldrich, catalog number: 10028-24-7)
4. KCl (Sigma-Aldrich, catalog number: 7447-40-70)
5. KH_2_PO_4_ (Sigma-Aldrich, catalog number: 7778-77-0)
6. Tween 20 (Sigma-Aldrich, catalog number: 9005-64-5)
7. Skim milk powder (FrieslandCampina)
8. DAPI (Sigma-Aldrich, catalog number: 28718-90-3)
9. ProLong^TM^ Gold antifade mountant (ThermoFisher Scientific, catalog number: P10144)
10. Nail polish

**Solutions**

1. Phosphate-buffered saline (PBS) (see Recipes)
2. Phosphate-buffered saline containing 0.05% tween (PBST) (see Recipes)
3. PBST + 5% milk (see Recipes)

**Recipes**

1. **PBS 10×**
  **Table:** 

Reagent	Final concentration	Amount
NaCl	N/A	80 g
Na_2_HPO_4_·2H_2_O	N/A	15 g
KCl	N/A	2 g
KH_2_PO_4_	N/A	1.2 g
Distilled water	N/A	up to 1 L
Total	10×	1 L
2. **PBST**
  **Table:** 

Reagent	Final concentration	Amount
Tween 20	0.05%	0.5 mL
PBS	1×	999.5 mL
Total	N/A	1 L
3. **PBST + 5% milk**
  **Table:** 

Reagent	Final concentration	Amount
Milk powder	5%	2.5 mg
PBST	1×	50 mL

## Equipment

1. Coverslip (Menzel-Glaser, catalog number: 631-1365)
2. ZEISS Axio Scan.Z1 (Carl Zeiss Microscopy GmbH, model: Axioscan 7)
3. Epredia^TM^ SuperFrost^TM^ microscope slides, ground 90° (Thermo Fisher Scientific, catalog number: 12372098)
4. A-PAP pen liquid-blocker (immunopen) (Cosmo Bio, model: DAI-APAP-R)
5. Straight tweezers
6. Cryostat (Leica Biosystems, model: CM3050 S)
7. Glass insert 70 mm wide for anti-roll systems (Leica Biosystems, catalog number: 14047742497)
8. Epredia^TM^ MX35 Premier^TM^ disposable low-profile microtome blades (Thermo Fisher Scientific, catalog number: 3052835)

## Software and datasets

1. ZEN 2 (Carl Zeiss, https://www.zeiss.com/microscopy/en/products/software/zeiss-zen.html )
2. Fiji (Schindelin et al. (2012), https://imagej.net/Fiji)
3. R v4.0.2 (R Core Team (2013), https://www.r-project.org/)
4. RStudio v1.3.959 (Allaire (2012), https://www.rstudio.com/ )
5. R scripts ( https://github.com/tabbassidaloii/ImageProcessing/blob/main/CapillaryDensity/Rscript )
6. Macros ( https://github.com/tabbassidaloii/ImageProcessing/tree/main/CapillaryDensity/Macros )

## Procedure

- **Cryosection of skeletal muscle biopsies**
  The procedure is detailed in Abbassi-Daloii et al. (2023a). In brief, this step entails the preparation of muscle biopsies for histology and immunofluorescence staining. Following the cleaning of equipment and temperature adjustments, the muscle biopsies are equilibrated inside the cryostat. Subsequently, biopsies are embedded in Tissue-Tek, placed on specimen holders, and cryosections of a specified thickness (10–16 μm) are collected onto SuperFrost slides. Store slides at -20 °C or -80 °C prior to immunostaining.
- **Immunofluorescence**
  This step describes immunofluorescence staining using antibodies for CD31 and laminin. Adding anti-CD105 as a second marker for endothelial cells is optional. The antibodies are prepared in PBST.
  1. Air dry slides from -20 °C for 30 min at room temperature (RT).
  2. Outline each section with an immunopen approximately 2–3 mm from the tissue edge. This reduces the required volume of the antibody mix.
*Note: Do not draw the line too close to the muscle sections as it can introduce artifacts in the image processing step.*
  3. Wash the sections in PBST.
  4. Blocking: Cover each section with PBST + 5% milk (~40 μL) for 30 min at RT.
  5. Wash the slides three consecutive times with a large volume of PBST (~40 μL), each time for 5 min.
  6. Primary antibody incubation: Cover each section with 20 μL of antibody mix containing anti-human CD31-Alexa Fluor^®^ 594-conjugated, rabbit anti-laminin, and anti-human CD105 biotin-conjugated (optional). Incubate for 2 h at RT.
*Note: Keep slides in the dark from this step onwards.*
  7. Wash the slides three consecutive times with an excessive volume of PBST, each time for 5 min.
  8. Secondary antibody incubation: Incubate sections with 20 μL of mixture of the following secondary antibodies for 1 h at RT: goat anti-rabbit Alexa Fluor^®^ 750-conjugated to detect anti-laminin and Streptavidin-Alexa Fluor^®^ 647-conjugated to detect anti-CD105 (optional).
  9. Wash the slides three consecutive times with an excessive volume of PBST, each time for 5 min.
  10. Nuclei counterstain is carried out by a short incubation (5–10 min) of the section with a DAPI solution (1:1,000 dilution in PBST, ~20 μL per section) in a dark environment. Afterward, gently rinse the sections with PBST to remove excess DAPI solution. DAPI binds to nucleic acids and stains the chromatin.
  11. Mounting: Cover the sections with ProLong^TM^ Gold antifade mountant (~10 μL per section). Cover the slide with a coverslip and fix it with nail polish.
*Note: Avoid any air bubbles on the sections as they will affect the image acquisition.*
  12. Keep for 24 h at RT in the dark prior to imaging.
  13. Store slides at 4 °C prior to imaging.
*Note: Slides can be kept at 4 °C for one month but imaging a week after immunostaining is preferable.*
- **Image acquisition**
  The image acquisition is detailed in Abbassi-Daloii et al. (2023a). In brief, we utilized a Zeiss Axio Scan.Z1 slide scanner with the ZEN 2 software. Per fluorophore, exposure and intensity were determined to maximize signal-to-noise ratio without bleaching. Imaging was carried out with a 10×/0.45 Plan-Apochromat objective. For high-throughput imaging, we recommend image acquisition with a slide scanner with a stitching option.
*Note: It is crucial to optimize the imaging settings on a test slide to determine the appropriate exposure time and intensity for each fluorophore. Adjustments of exposure time and focusing algorithms may affect the visibility of the fluorophore signal. To achieve the best signal-to-noise ratio without causing bleaching, it is necessary to optimize the intensity and exposure time for each fluorophore/channel.*
  For all channels, utilize single band filters with the following excitation ranges:
  - Channel 1 (DAPI): 335–355 nm excitation
  - Channel 2 (CD31, Alexa Fluor^®^ 594): 574–599 nm excitation
  - Channel 3 (CD105, Alexa Fluor^®^ 647): 650–670 nm excitation (optional)
  - Channel 4 (Laminin, Alexa Fluor^®^ 750): 672–747 nm excitation
*Notes:*
  1. *It is crucial to maintain consistent image acquisition settings for all slides throughout different batches. This ensures uniformity and allows for reliable comparisons between samples.*
  2. *To eliminate batch effect, we recommend staining all samples in one batch and then imaging all slides in one session. When staining samples in multiple batches, it is important to conduct imaging in the same order of batches. This consistency ensures that the time interval between staining and imaging remains constant across all batches, promoting accurate and comparable results.*
  3. *When employing the Axio Scan.Z1 slide scanner, each slide's output will be in the Carl Zeiss Image format (CZI) dataset, containing an image for each section of the slide.*
- **Image processing**
  1. Laminin segmentation and object quantification
    The procedure for converting image format and laminin segmentation is carried out in ImageJ/Fiji, using five sequential macros. The macros are found in: ( https://github.com/tabbassidaloii/ImageProcessing/tree/main/CapillaryDensity/Macros/), macros 2–4. The macros are fully automatic, besides macro number 3, which might require a manual adjustment (as explained in the macro and in Abbassi-Daloii et al., 2023a). The outputs from the five macros are collected in a folder named “check” with mask images after each step, and a folder “ROI” with .txt files reporting the area of the segmented laminin objects that will be used for the calculation of capillary density.
    An example of laminin staining and segmentation is in Figure 2.
    **Figure 2.**
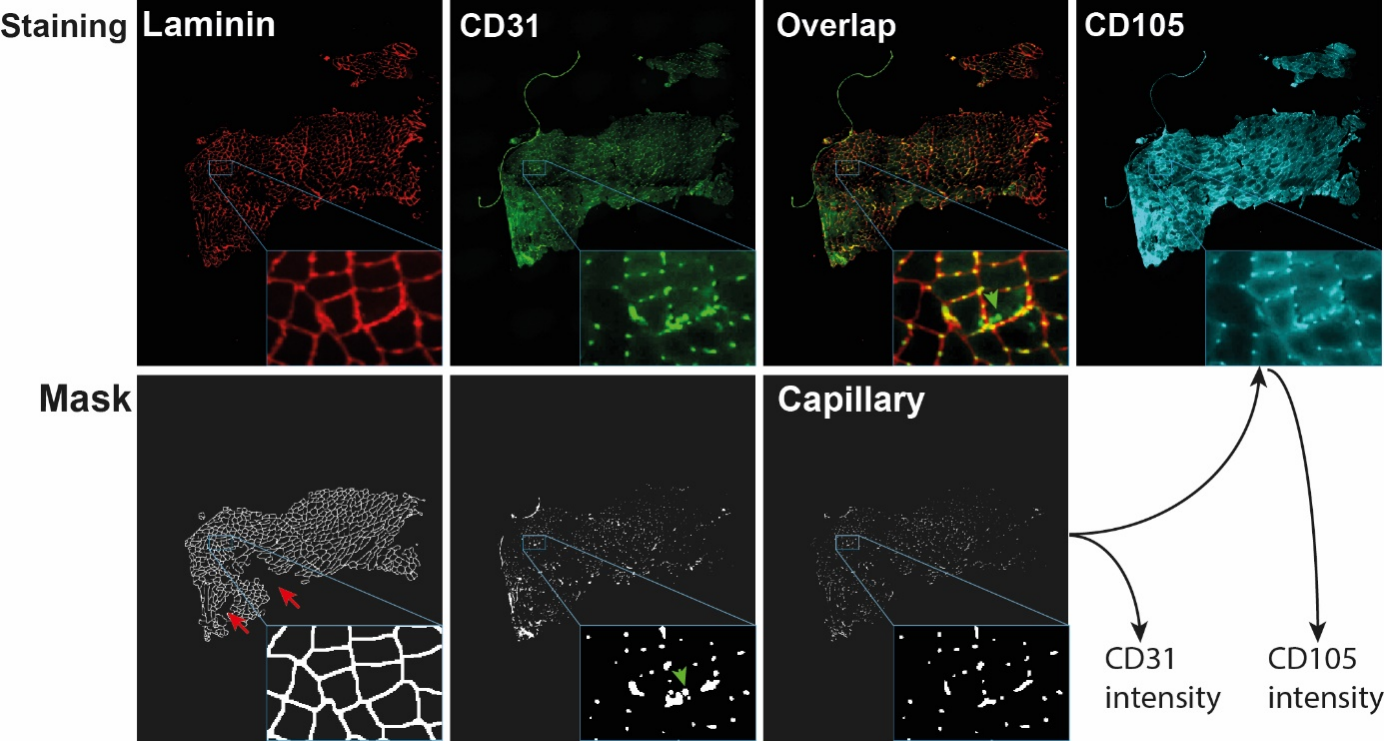
Visualization of capillary mask and capillary output generation. The images show the entire cross section with a zoom-in insert in the right bottom of each image. Red arrows point to laminin regions that were excluded from the mask after segmentation. Green arrows point to CD31 objects that did not overlap with laminin and were therefore excluded from the capillary mask. The capillary mask is used to obtain CD31 intensity and CD105 intensity.
  2. Segmentation and quantification of CD31 and CD105 objects
    This step is executed using macro number 6. We perform the segmentation of the CD31 signal and compute the intersection with the laminin segmentation; these objects are considered as capillary. The output files contain the mean fluorescence intensity, area, and circularity of the capillary objects for both CD31 and CD105.
    - A mask is made on CD31 images using a Gaussian Blur filter to reduce noise, resulting in a smoother image.
    - Thresholding is applied using the "Li dark" algorithm to convert the CD31 channel into a binary image that distinguishes between foreground and background pixels.
    - The Watershed algorithm is then utilized to accurately separate cells that may be touching or overlapping.
    - The Fill Holes algorithm is employed to fill any empty spaces within the segmented regions.
    - The laminin segmentation mask is added to identify the overlap between laminin and CD31 objects, resulting in a *capillary mask*.
    - Once the capillaries have been segmented, the mean fluorescence intensity is measured in the original CD31 and CD105 channels.
    - The intensity, circularity, and area of CD31 and CD105 are in the output file that is saved in a folder named “segmentation.” An example of CD31 and CD105 staining, CD31 segmentation, and capillary mask are in Figure 2 .
*Note: Steps outlined previously can be conveniently executed by running two Windows Batch Files available on GitHub. These files are specifically designed to automate the process, allowing for a streamlined and efficient implementation of the protocol.*

*
https://github.com/tabbassidaloii/ImageProcessing/tree/main/CapillaryDensity/Macros/BatchFiles*

- **Capillary density quantification**
  In this step, we assess the filtering procedures and quantify capillary density. These analyses are carried out in the RStudio software along with the R statistical software. To facilitate the execution of these steps, we have made the R Markdown file accessible on GitHub: https://github.com/tabbassidaloii/ImageProcessing/blob/main/MyofiberTyping/Rscript/CapillaryDensity.Rmd Within the following steps, we indicate the specific R code chunk within this R markdown file that should be utilized for each task.
  1. Calculate the total myofiber area:
    - Consolidate myofiber data from all samples by running the "myofibersTotalArea" R code chunk.
    - Count the number of segmented objects (myofibers) per sample.
    - Compute the total myofiber area per sample.
    - Retain the replicate with the highest number of myofibers.
    - Exclude samples with a small number of myofibers (<100).
  2. Calculate the total CD31 positive area:
    - Consolidate CD31 and CD105 quantification data from all samples by running the "positiveCD31Area" R code chunk.
    - Compute and plot the total proportion of CD31 positive area per sample.
  3. Calculate capillary density:
    - Apply filtering for capillaries by running “capillaryDensity” R chunk code:
      • Select objects with positive signals for both CD31 and CD105.
      • Choose objects larger than 3 µm^2^ and smaller than 51 µm^2^.
      • Include objects with circularity larger than 0.5.
    - Compute and plot capillary density as the number of capillaries per unit (µm) of the total myofiber area.
*Note: The capillary density can be alternatively calculated as the number of capillaries per myofibers, which are computed in the "myofibersTotalArea" R code chunk.*

## Validation of protocol

The procedures outlined in this protocol have been used in the following research article: Abbassi-Daloii et al. (2023b) (Figure 4).

## General notes and troubleshooting

**General notes**

1. This procedure requires a basic understanding of immunofluorescence, fluorescence imaging, image processing in ImageJ, and data analysis in R.
  - For basics in immunofluorescence, please refer to the following paper: Im et al. (2019).
  - For basics in fluorescence imaging, you can refer to the following source: Ogundele et al. (2013).
  - The basics of using ImageJ can be found in the "ImageJ User Guide" document, available at: https://imagej.nih.gov/ij/docs/guide/user-guide.pdf.
2. If you encounter problems running the macros, please contact us.
3. The definition of capillary objects is based on three markers: CD31 & laminin overlap and overlap with CD105. To reduce costs, it is possible to omit CD105. In our experience, CD31 staining was more specific compared with CD105.
4. When assessing changes in capillary density between tissues or pathological conditions, aim for at least 1,000 capillary masks per sample.
5. While this procedure is described for human skeletal muscle cross sections, it can be applied to animal models (i.e., mouse, rat). The antibodies we specify should be tested for cross-reactivity in other models. We recommend testing the antibodies before conducting a large experiment.
